# Disentangling Orbital and Confinement Contributions to *g*‑Factor in Ge/SiGe Hole Quantum Dots

**DOI:** 10.1021/acs.nanolett.6c00999

**Published:** 2026-06-17

**Authors:** L. Sommer, I. Seidler, F. J. Schupp, S. Paredes, N. W. Hendrickx, L. Massai, K. Tsoukalas, A. Orekhov, E. G. Kelly, S. W. Bedell, G. Salis, M. Mergenthaler, P. Harvey-Collard, A. Fuhrer, T. Ihn

**Affiliations:** † IBM Research Europe - Zürich, Säumerstrasse 4, 8803 Rüschlikon, Switzerland; ‡ IBM Quantum, T. J. Watson Research Center, 1101 Kitchawan Road, Yorktown Heights, New York 10598, United States; § Solid State Physics Laboratory, ETH Zürich, 8093 Zürich, Switzerland

**Keywords:** Germanium quantum dots, Excited-state spectroscopy, Coulomb blockade addition spectroscopy, Magnetic-field dependence, Germanium qubits, g-factor determination

## Abstract

Spin qubits are typically operated in the lowest orbital of a quantum dot to minimize interference from nearby states. In valence-band hole systems, strong spin–orbit coupling links spin and orbital degrees of freedom, strongly influencing the hole *g*-factor, a key parameter for qubit control. We investigate the out-of-plane *g*-factor in Ge quantum dots using excitation (single-particle) and addition (many-body) spectra. Excitation spectra allow us to distinguish the pure Zeeman *g*-factor from orbital contributions to the magnetic field splitting of states despite the strong spin–orbit coupling. This distinction clarifies discrepancies between *g*-factors extracted with the two methods, for different orbital states and different hole numbers. Furthermore, we find gate-tunability of *g*-factors at the level of 15%, highlighting its relevance for all-electric qubit manipulation.

## Graphical abstract

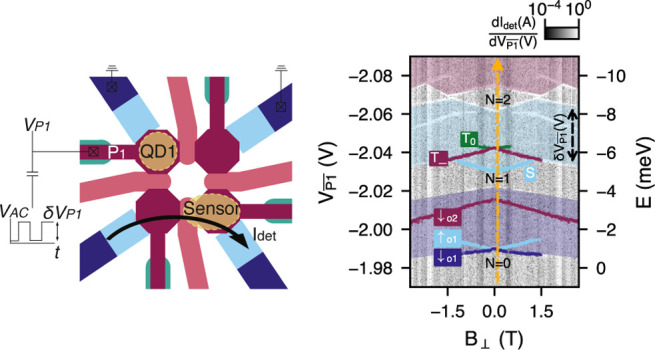

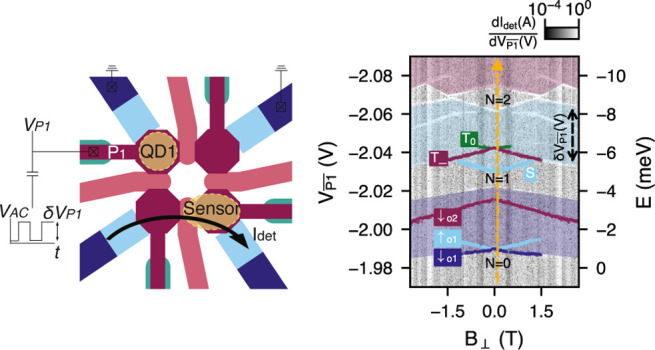

Spin qubits in semiconductor quantum dots are typically operated in the lowest orbital to reduce interference from nearby states. In hole-based systems, strong spin–orbit interaction couples spin and orbital degrees of freedom, significantly influencing the hole *g*-factor, a key parameter for qubit control. Precise knowledge of *g*-factors is therefore essential for reliable qubit operation.

Ge/SiGe heterostructures provide a favorable platform for hole quantum dots. Their low effective mass extends the wave function, easing gate design constraints.^1^ In germanium, the g-tensor is strongly anisotropic.^2–5^ In heterostructures with identical alloy composition, quantum well thickness, and buffer thickness, the out-of-plane *g*-factor typically ranges from 10 to 12,^2^ while the in-plane *g*-factor spans approximately from 0.01 to 1 ^2,5–7^ depending on magnetic field orientation. Current theory captures the general magnitude of these *g*-factors^8^ but fails to reproduce their angular dependence or precise values, likely due to orbital structure, confinement potential, or local strain.

Probing how *g*-factors vary with hole number and orbital state is important for scaling qubit systems. Gate-defined quantum dots enable magnetospectroscopic measurements prior to qubit operation. Coulomb blockade addition spectroscopy (CBAS) probes many-body energy differences between successive hole occupation numbers.^9^ In this technique, Coulomb screening and exchange interactions induce additional orbital level shifts that, together with Zeeman-splitting, modify the apparent *g*-factor. Pulsed excited-state spectroscopy (PESS), in contrast, reveals the excitation spectrum at fixed occupancy,^10–12^ thereby avoiding occupation-dependent orbital shifts, and enabling a more direct access to the pure Zeeman splitting. At the same time, it allows the extraction of orbital level shifts for states that share the identical spin quantum numbers in an applied magnetic field. Comparing these methods within the same dot enables a clear separation of Zeeman contributions from orbital and confinement-induced effects, thereby helping to resolve discrepancies between the two approaches. Throughout this work, orbital effects refer to differences in the magnetic-field dependence of two states in the one-hole regime that share the same spin orientation but differ in their orbital wave function. At higher hole occupancies, additional shifts arise from screening and exchange-induced modifications of the confinement potential, which we refer to as confinement effects.

In this study, we investigate a gate-defined quantum dot in a planar Ge/SiGe heterostructure using an integrated charge sensor. We measure energy spectra of the first few hole states using CBAS and PESS, extract Zeeman splittings, and compare *g*-factors obtained from addition and excitation spectra. Such a comparison has previously been reported only for silicon.^12^ By tuning the confinement potential with gate voltages, we also explore gate-induced modifications of the *g*-factor, demonstrating tunability relevant for all-electric qubit control.

Measurements were performed on two double quantum dot (DQD) devices, referred to as device 1 and device 2, to assess the reproducibility of the experimental findings. These devices, fabricated from the same wafer in different fabrication runs, were nominally identical. The dots were formed within the 20 nm thick quantum well of a strained Ge/SiGe heterostructure.^2,13^ In each device, two quantum dots are located beneath plunger gates P_1_ and P_2_, with the tunable interdot coupling controlled by gate B_12_ (Figure 1a). A nearby quantum dot charge sensor enables the detection of charge transitions. This technique allows us to probe the first few-hole states when the tunnel rate between the quantum dot and the reservoir is too low for direct transport measurements. Using virtual gate voltages $V_{\overset{®}{P1}}$ and $V_{\overset{®}{P2}}$ , we independently control the charge occupancy (*N*, *M*) of each quantum dot (Figure 1b) (see Supporting Information S1 for details). The devices are operated in a regime (*N*, 0) where quantum dot 2 (QD2) is fully depleted, and only quantum dot 1 (QD1) is populated. The occupancy of QD1 is tuned from *N* = 0 to 8 holes along the arrow in Figure 1b.

**Figure 1:**
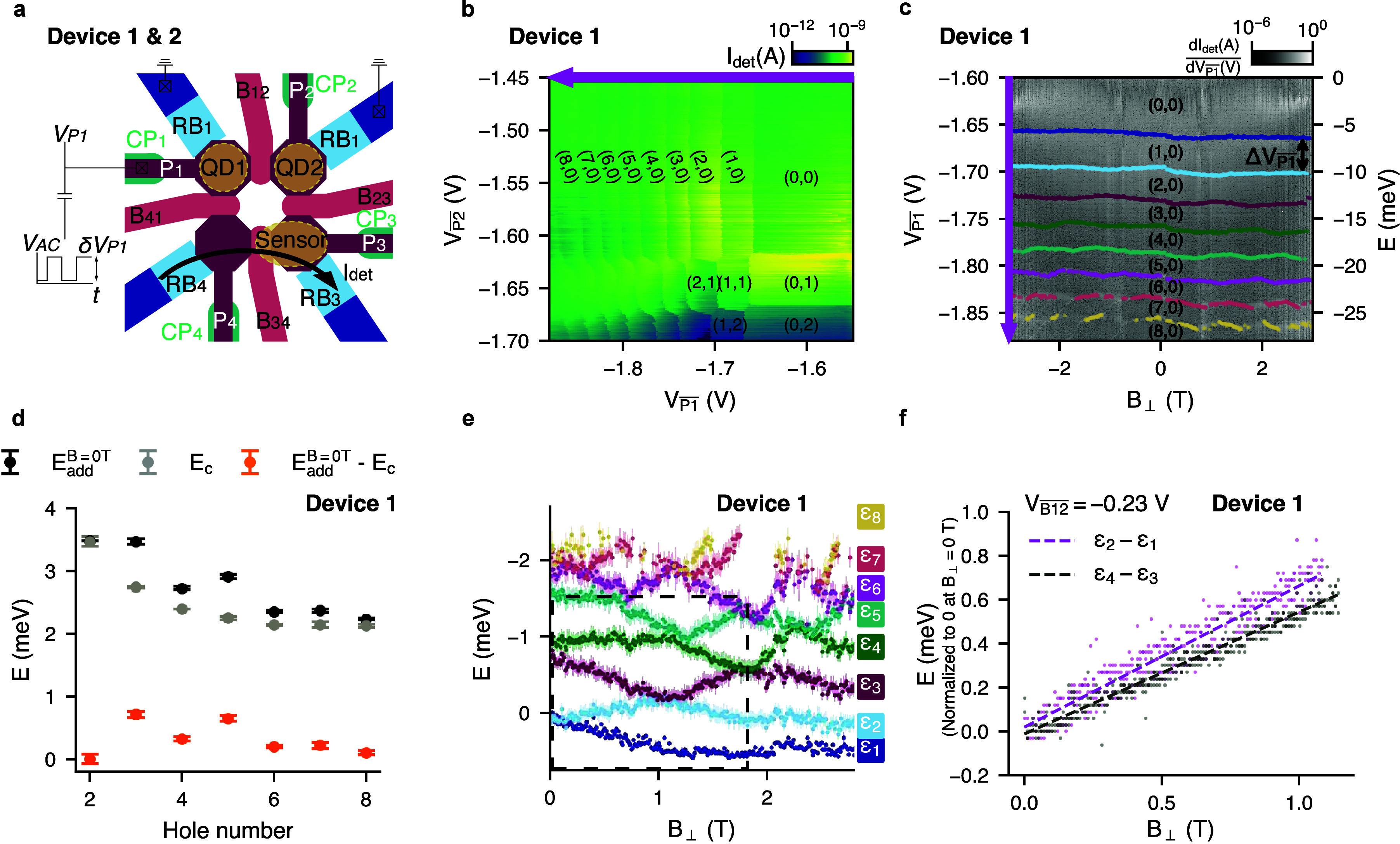
Operation of the double quantum dot (DQD) system and Coulomb blockade addition spectroscopy. (a) Schematic of the device. The DQD is defined beneath plunger gates P_1_ and P_2_ and is monitored via a proximal charge sensor. Interdot coupling is tuned via the central barrier gate B_12_, while coupling to the reservoirs is controlled by gates RB_1_ and RB_2_. The charge sensor signal is extracted from the differential current between source and drain, *I*_SD_, as indicated by the black arrow. (b) Charge stability diagram of the DQD as a function of virtual plunger gates $V_{\overset{®}{P1}}$ and $V_{\overset{®}{P2}}$ . Charge configurations are labeled as (*N*, *M*), denoting the hole occupation of each dot. (c) Magnetospectroscopy of Coulomb blockade addition energies, revealing spin and orbital structure. Charge states are again labeled as (*N*, *M*). (d) Extracted addition energies (black), charging energies (gray), and single-particle energies (orange) as a function of total dot occupation. Error bars are smaller than the marker size. (e) Single-particle energy spectrum obtained by subtracting the charging energy *E*_*C*_ from the addition energies in panel (d). The dashed box highlights the region analyzed in Figure 2b. (f) Linear fit to the spin-split energy levels used to extract the CBAS *g*-factor, based on the slope prior to the first level crossing.

To reconstruct the energy spectrum of QD1, CBAS is performed on device 1 as a function of magnetic field *B*_⊥_ applied perpendicular to the quantum well plane (see Figure 1c). The plunger gate voltage $V_{\overset{®}{P1}}$ is swept toward more negative voltages (arrow in Figure 1c) at fixed values of *B*_⊥_, and the derivative $dI_{det}/dV_{\overset{®}{P1}}$ is plotted normalized to its maximum value. The reverse sweep directions are also recorded but not shown. After each pair of sweeps, *B*_⊥_ is incremented, and the charge sensor is retuned to optimize sensitivity to the first charge transition. Charge transitions are identified by locating peaks in $dI_{det}/dV_{\overset{®}{P1}}$ , as a function of $V_{\overset{®}{P1}}$ for every *B*_⊥_. These peak positions, marked by colored lines in Figure 1c, delineate the charge states indicated between them.

The addition energy for each hole number *N* is extracted from the spacing $\Delta V_{\overset{®}{P1}}$ between adjacent peaks in Figure 1c using

$$E_{add}\left(N\right)=\mu_{N+1}-\mu_{N}=e\alpha_{\overset{®}{P1}}\Delta V_{\overset{®}{P1}}$$

with a gate lever arm $\alpha_{\overset{®}{P1}}=0.1$ (see Supporting Information for details on S2 spectrum and S3 lever arm extraction). Figure 1d shows these addition energies at *B*_⊥_ = 0 T for the first eight holes (black points). Superimposed on a generally decreasing trend, we observe enhanced addition energies for adding the third and the fifth hole (hole numbers 2 and 4, respectively, in Figure 1d). This enhancement is a manifestation of the occupation of higher-energy orbital states in the dot, once the low-energy 2-fold degenerate orbitals have been occupied by spin-up and spin-down holes.

To obtain an approximate magnetic-field-dependent single-particle spectrum ϵ_*N*_(*B*_⊥_) (*N* = 1, 2, ...) from the raw data (Figure 1c), we start from

$$E_{add}\left(N,B_{⊥}\right)=E_{C}\left(N\right)+\Delta \epsilon_{N}\left(B_{⊥}\right)$$

where *E*_C_(*N*) is the magnetic field independent charging energy for the dot with *N* holes, and Δϵ_*N*_(*B*_⊥_) = ϵ_*N*+1_(*B*_⊥_) – ϵ_*N*_(*B*_⊥_) is the magnetic field dependent spacing of excited states [^14^]. We determine *E*_C_(*N*) as the energy offset such that ${min}_{B_{⊥}}\left[\Delta \epsilon_{N}\left(B_{⊥}\right)\right]=0$ for one value of *B*_⊥_ for neighboring levels ϵ_*N*_, as shown in Figure 1e. To define the energy reference, an offset is applied such that ϵ_1_(*B*_⊥_ = 0 T) = 0.

Before discussing the details of Figure 1e, we plot the extracted values of *E*_C_(*N*) as gray points in Figure 1d. The figure shows that the charging energy decreases monotonically with increased hole number, in contrast to the addition energy *E*_add_(*N*). This effect is due to increased dot size, i.e. increased dot capacitance, driven by Coulomb interaction. This procedure leaves us with values Δϵ_*N*_(*B*_⊥_) = *E*_add_(*N*, *B*_⊥_) – *E*_C_(*N*), which are plotted for *B*_⊥_ = 0 T as orange points in Figure 1d. These points reflect the alternating spin-filling of orbital levels mentioned before, with an enhanced Δϵ_*N*_ for adding the third and fifth hole.

Returning to the spectrum ϵ_*N*_(*B*_⊥_) in Figure 1e, we see that the 2-fold degenerate levels for *N* = 1, 2 and *N* = 3, 4 split linearly for small *B*_⊥_ < 1 T. We interpret this splitting as an effective Zeeman splitting and determine the out-of-plane CBAS *g*-factor using

1 $$g^{\epsilon_{N+1}-\epsilon_{N}}=\frac{1}{\mu_{B}}\frac{d\left(\Delta \epsilon_{N}\left(B_{⊥}\right)\right)}{dB_{⊥}}$$

where the derivative is obtained from a linear fit to the Zeeman splitting (see Figure 1f), and μ_*B*_ is the Bohr magneton. The extracted values are summarized in Table 1. The CBAS *g*-factor for the first spin pair, $g^{\epsilon_{2}-\epsilon_{1}}$ , agrees well by 2% with values obtained from qubit spectroscopy^2^ via Pauli spin blockade readout. In contrast, the second spin pair, $g^{\epsilon_{4}-\epsilon_{3}}$ , exhibits a reduced *g*-factor, consistent with previous observations in ref 10. In this reference, the observed reduction of $g^{\epsilon_{4}-\epsilon_{3}}$ as compared to $g^{\epsilon_{2}-\epsilon_{1}}$ is attributed to mechanisms such as heavy-hole–light-hole mixing, leakage of the wave function into the SiGe barrier, or hole–hole interactions. The CBAS method reproduces the well-known strongly anisotropic *g*-tensor when the magnetic field orientation relative to the sample plane is changed (data shown in Supporting Information S10, Figure S15).

**Table 1:** Extracted Absolute *g*-Factors with 3*σ*-Error Bars from CBAS and PESS for Both Devices, Where N is the Transition Hole Number^a^

	method		*N*	#orbital
**Device 1**				
$g^{\epsilon_{2}-\epsilon_{1}}$	CBAS	11.12(63)	1, 2	1
$g^{\epsilon_{4}-\epsilon_{3}}$	CBAS	9.60(36)	3, 4	2
**Device 2**				
$g^{\epsilon_{2}-\epsilon_{1}}$	CBAS	11.25(33)	1, 2	1
*Bare Spin*				
$g^{{↑}_{o1}-{↓}_{o1}}$	PESS	9.86(15)	1	1
$g^{T_{0}-T_{-}}$	PESS	10.25(63)	2	2
*Spin and Orbital*				
$g^{{↑}_{o1}-{↓}_{o2}}$	PESS	13.71(33)	1	1, 2
$g^{S-T_{-}}$	PESS	14.25(33)	2	1, 2

a Additionally, the orbital number investigated in the analysis are indicated.

While CBAS provides an approximate single-particle level spectrum ϵ_*N*_(*B*_⊥_), it can be expected that this spectrum is not described by a single-particle model Hamiltonian incorporating a fixed in-plane confinement potential, such as a Fock–Darwin model (see Supporting Information S4) or a similar model based on the Luttinger–Kohn Hamiltonian. For example, gate voltage sweeps alter the confinement potential, modifying the spectrum parametrically. In addition, screening effects further modify the effective confinement potential, and exchange and correlation effects can be relevant. The CBAS procedure may also mask spin–orbit interaction effects that have been predicted to lead to avoided level crossings.^15^

To directly probe the excited-state energies, we performed PESS measurements on device 2. To this end, we first convinced ourselves that the addition spectrum of this device (see Figure 2a) shows qualitative agreement with device 1 (see Figure 1e). The dashed rectangle in both figures highlights the corresponding voltage range in the two devices, where we focus on the first three energy levels. The extracted CBAS *g*-factor $g^{\epsilon_{2}-\epsilon_{1}}$ of device 2 is comparable to that of device 1 (see Table 1 and Figure 2b). Additionally, Δϵ_2_ is similar in both devices, ensuring consistency throughout the experiment. In both spectra Figure 1e and Figure 2a, discontinuities are visible at the level crossings, highlighted with the dashed line in Figure 2a. It seems to be a multiparticle effect and is further discussed in Supporting Information S5.

**Figure 2:**
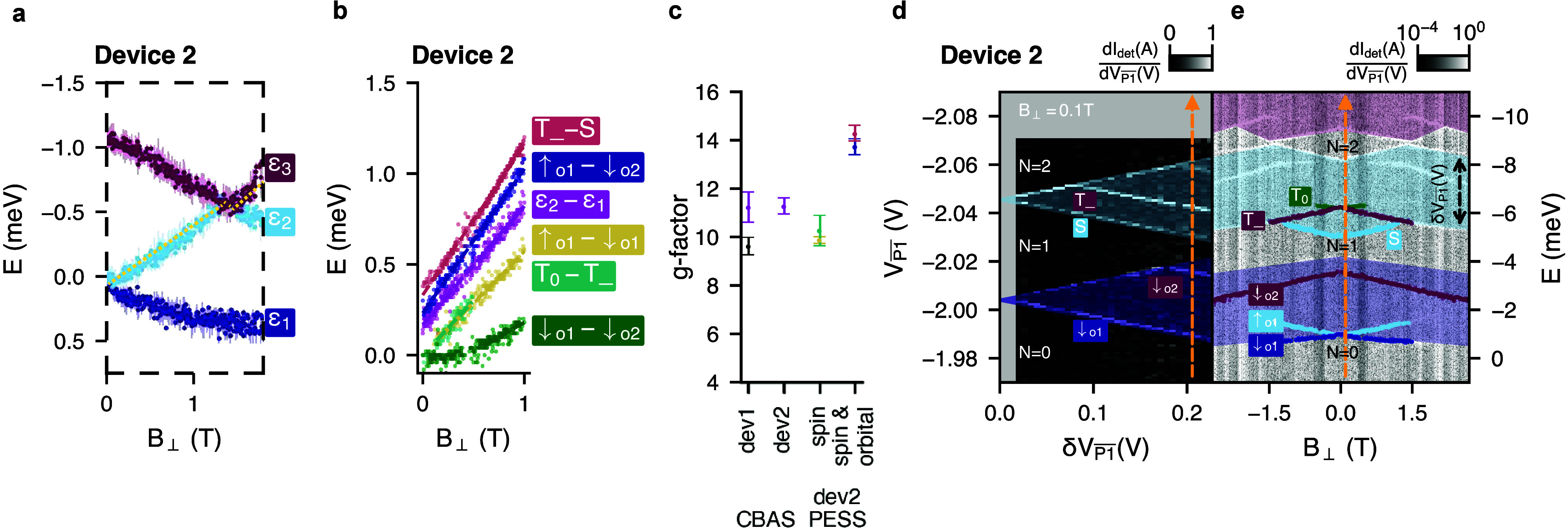
Comparison of CBAS analysis to PESS. (a) Energy spectrum obtained via CBAS. Ground-states of **e** from magnetospectroscopy are analyzed analogously. The yellow line marks the discontinuity at the intersection of ϵ_2_ and ϵ_3_. (b) Linear fits to the spin-split energy levels used to extract the CBAS and PESS *g*-factor. All data are shown in absolute values. For clarity, an offset is added to all traces except those corresponding to pure spin transitions (*↑*_o1_ – *↓*_o1_ and *T*_0_ – *T*_–_). (c) CBAS and PESS *g*-factor for both devices with 3σ-error bars. (d) Derivative of the charge sensor current, $dI_{det}/dV_{\overset{®}{P1}}$ , plotted as a function of pulse amplitude *δV*_P1_ and DC gate voltage $V_{\overset{®}{P1}}$ at *B*_⊥_ = 0. Colored regions indicate where specific hole numbers can be dynamically loaded in response to the applied pulses. (e) $dI_{det}/dV_{\overset{®}{P1}}$ as a function of $V_{\overset{®}{P1}}$ and *B*_⊥_ at fixed $\delta V_{\overset{®}{P1}}$ . The hole number *N* is labeled, and regions sensitive to excited-state spectra are highlighted. Extracted energy levels are overlaid as colored lines.

In PESS following refs 12 and 16 , rectangular virtualized AC pulses of amplitude $\delta V_{\overset{®}{P1}}$ , frequency *f* = 20 kHz, and 50% duty cycle are superimposed on the DC gate voltage $V_{\overset{®}{P1}}$ . This periodic modulation shifts the dot levels relative to the reservoir’s Fermi level. The tunnel rates between the dot and the reservoir are measured by varying the pulse frequency, confirming that the tunnel rates are below 1 kHz, the minimum of the measurement range (see Supporting Information S6). An integration time of 40 ms is used to optimize the visibility of excited-states. Two branches appear as the pulse amplitude is increased corresponding to the edges of the two-level pulse (Figure 2d). For amplitudes $e\alpha_{\overset{®}{P1}}\delta V_{\overset{®}{P1}}$ exceeding an excitation energy at fixed hole number, additional lines appear parallel to the lower branch edge. Such excited states are observed both when filling the *N* = 1 state in the transition region *N* = 0 ↔ 1 region (purple) and *N* = 2 in the *N* = 1 ↔ 2 region (blue) in Figure 2d.

Measuring PESS at a fixed pulse amplitude $\delta V_{\overset{®}{P1}}=0.2V$ indicated by the dashed arrow in Figure 2d as a function of $V_{\overset{®}{P1}}$ and *B*_⊥_ results in the raw spectrum shown in Figure 2e. In the *N* = 0 ↔ 1 region, we observe the 2-fold degenerate ground state at *B*_⊥_ = 0 T, that Zeeman-splits into two levels (purple, *↓*_o1_ and blue *↑*_o1_) at finite *B*_⊥_ giving rise to the pure spin PESS *g*-factor $g^{{↑}_{o1}-{↓}_{o1}}$ shown in Table 1. The first excited orbital state (red *↓*_o2_) runs nearly parallel to the ground state, suggesting that it shares the same spin projection. However, its Zeeman splitting is not visible in our measurements, likely due to insufficient difference in tunnel rates. While the excited state *↓*_o2_ changes the tunnel rate sufficiently to produce a contrast in the averaged measurement, its Zeeman split partner *↑*_o2_ does not alter the tunneling dynamics sufficiently to be detected. In the *N* = 1 ↔ 2 region, the ground state (singlet state, blue *S*) is nondegenerate with total spin angular momentum 0. The observed triplet states *T*_–_ and *T*_0_ involve a second excited orbital state, but are energetically lowered by exchange, placing them well below the single-particle orbital splitting observed for *N* = 0 ↔ 1 $(\Delta {\mathrm{orb}}_{N_{0↔1}}^{B_{⊥}=0T}=2.56\;meV$ , $\Delta {\mathrm{orb}}_{N_{1↔2}}^{B_{⊥}=0T}=1.07\;meV)$ ). Additionally, the confinement potential is increasingly screened when adding more holes, which also reduces this splitting. The triplet states Zeeman-split linearly into *T*_–_ and *T*_0_ at finite *B*_⊥_ giving rise to the PESS *g*-factor $g^{T_{0}-T_{-}}$ in Table 1. An additional excited state at higher energy is also observed,^17^ which is beyond our interest in this paper. At the onset of the *N* = 2 ↔ 3 region, we identify the ground-state with the total angular momentum of the *N* = 1 ground-state. Overall, the data suggest that states fill in a sequence of alternating sign of angular momentum *z*-projection.

Figure 2b shows a direct comparison of the energy level differences from CBAS and PESS measurements used to determine the CBAS and PESS *g*-factors in Table 1. The excited-state pairs highlighted in Figure 2e allow extraction of *g*-factors using the slope-based method of the linear splitting with magnetic field as described earlier. The values, shown in Figure 2c (here we show 3σ-error bars) and summarized in Table 1, reveal that $g^{{↑}_{o1}-{↓}_{o1}}$ (yellow) is reasonably close to $g^{T_{0}-T_{-}}$ (turquoise) within experimental error, where both are pure spin *g*-factors. This contrasts with the CBAS *g*-factor (purple) which is significantly larger, highlighting the fact that the chosen spectroscopy method can have an important influence on the *g*-factor.

Interestingly, the reduction of the CBAS $g^{\epsilon_{4}-\epsilon_{3}}$ as compared to $g^{\epsilon_{2}-\epsilon_{1}}$ , discussed before, is not observed when comparing the corresponding PESS $g^{T_{0}-T_{-}}$ to $g^{{↑}_{o1}-{↓}_{o1}}$ .

To investigate the orbital contribution to the *g*-factors within the same charge configuration, we compute the energy difference between the first excited orbital state (red, *↓*_o2_) and the ground orbital state (purple, *↓*_o1_), and plot this difference in green in Figure 2b. This difference remains approximately constant up to 0.5 T, after which it begins to increase. By comparing the pure Zeeman level splitting (*↓*_o1_ – *↑*_o1_) in the range of zero to one Tesla to the change in orbital level splitting between ground and first excited state (*↓*_o1_ – *↓*_o2_), we estimate that changes in orbital wave function can contribute up to 10% to the apparent Zeeman splitting measured between spin states with distinct orbital wave functions, such as *S* – *T*_–_. The nonlinearities caused by such orbital effects appear as systematic deviations of linear fits from the data (see Supporting Information Figure S6e). Beyond this experimental evidence for orbital influences on the *g*-factor determination, we confirm a similar order of magnitude from simple theoretical considerations (Supporting Information S7). Based on these findings, when orbital states are included in the PESS *g*-factor extraction, the orbital contribution appears to add to the bare spin component resulting in an overall increase in the PESS *g*-factor for $g^{{↑}_{o1}-{↓}_{o2}}$ (*N* = 0 ↔ 1) and $g^{S-T_{-}}$ (*N* = 1 ↔ 2), as summarized in Table 1. Similar to CBAS, PESS can also be used to measure the anisotropic *g*-tensor (see Supporting Information S10, Figure S16).

Having established the key differences between the two methods for *g*-factor determination, we now examine the voltage tunability of the *g*-factor. Such a tunability would greatly enable gate-driven qubit operation.^18,19^ By varying the voltages applied to adjacent gate electrodes, we track the evolution of the *g*-factor using both CBAS and PESS measurement techniques. For device 1, we investigate the tunability of the CBAS *g*-factor by varying the interdot barrier gate voltage $V_{\overset{®}{B12}}$ from – 0.23 V to – 0.35 V (the charge stability diagrams can be found in Supporting Information S8). This voltage range is chosen to avoid hysteretic gate response inherent to the device.^13^ Figure 3a shows the approximate single-particle energies at *B*_⊥_ = 0 T for the first four levels. They remain nearly unchanged within experimental uncertainty (3σ-error bars). We interpret this as an indication that the confinement potential is primarily shifted in space without significantly altering its characteristic shape.

**Figure 3:**
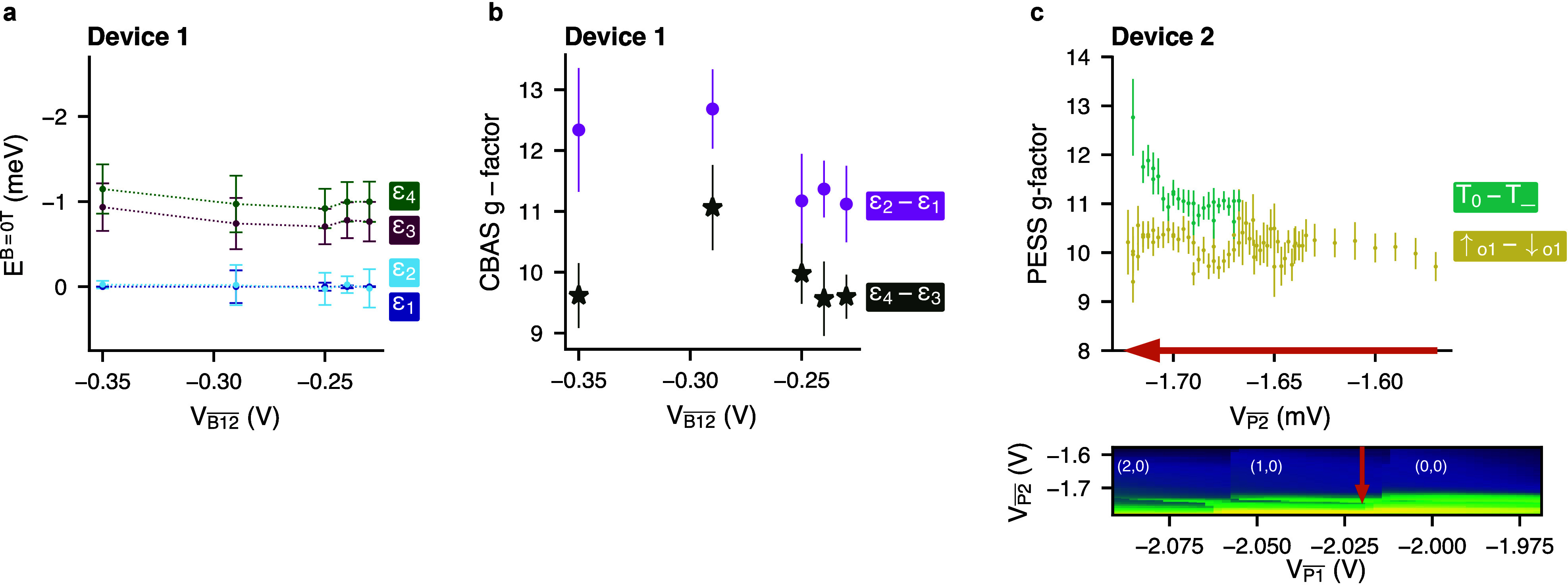
Voltage-tunable *g*-factor. (a) Single-particle energies at *B*_⊥_ = 0 T as a function of quantum dot occupation for different values of $V_{\overset{®}{B12}}$ . (b) CBAS *g*-factor extracted via CBAS as a function of the interdot barrier voltage $V_{\overset{®}{B12}}$ . (c) PESS *g*-factor as a function of the plunger gate voltage applied to the neighboring quantum dot. The lower plot shows the corresponding charge stability diagram, with arrows indicating the direction of plunger gate tuning. The charge configuration is labeled as (*N*, *M*).

Figure 3b shows the CBAS *g*-factors for the first two Zeeman-split pairs. Changes in the *g*-factor beyond experimental uncertainty are observed at the level of approximately 15% within the accessible gate voltage range. For comparison, recent qubit studies report variations of order 10% over smaller tuning ranges.^5^ The extracted CBAS *g*-factor $g^{\epsilon_{2}-\epsilon_{1}}$ exhibits a more consistent increase for increasingly negative $V_{\overset{®}{B12}}$ compared to $g^{\epsilon_{4}-\epsilon_{3}}$ . Unfortunately, changing the barrier gate $V_{\overset{®}{B12}}$ leads to a significant cross-talk reducing the tunnel coupling of the dot to the lead. This effect prohibited measurements of the *g*-factor using PESS, for which the tunnel rate must remain within the operational bandwidth of our electronics ( $∼10^{3}$ –10^7^ Hz). To still enable PESS *g*-factor measurements, we employ the virtual plunger gate $V_{\overset{®}{P_{2}\;}}$ instead. By sweeping the plunger gate of the neighboring dot toward the interdot regime, as indicated by the arrows in the bottom of Figure 3c, the quantum dot wave function gradually shifts from beneath $V_{\overset{®}{P1}}$ toward $V_{\overset{®}{P2}}$ . The extracted pure spin PESS *g*-factors are shown in Figure 3c (data for the second dot in Supporting Information S10). While $g^{{↑}_{o1}-{↓}_{o1}}$ remains constant within experimental uncertainty, $g^{T_{0}-T_{-}}$ varies approximately by 20% as the wave function delocalizes across the barrier and subsequently relocalizes in the second dot. This sensitivity likely arises from changes in the local potential and strain as the hole’s position shifts relative to the interdot barrier, consistent with previous reports on electrostatic and strain-induced *g*-factor variability.^5,18,20^

In conclusion, we have identified an experimental method to determine the pure Zeeman *g*-factor in p-type germanium quantum dots and to distinguish it from orbital contributions arising from the strong spin–orbit coupling in this material. The significant influence of orbital effects at the level of 10% of the bare Zeeman splitting in the magnetic field range between 0 and 1 T adds to explanations for the variations in the extracted *g*-factors found between different methods (CBAS vs PESS), across orbital states, and for different hole numbers. Additional influences are expected from changes in gate-induced changes to the confinement potential and from screening-, exchange- and correlation-effects relevant in dots of more than one hole. These findings highlight the complexity of an accurate characterization of *g*-factors in hole quantum dots and indicate that comparisons between *g*-factors obtained in the community need to be made with great care. With these insights in mind we have explored the opportunity to gate-control the *g*-factors. A tunability of up to 15% was found by shifting the quantum dot states spatially, which may indicate an opportunity for all-electric qubit manipulation.

## Supplementary Material



## Data Availability

The data and analysis that support the findings of this study are available in a Zenodo repository https://doi.org/10.5281/zenodo.20608426.

## Notes

Device fabrication and materials stack, experimental and cryogenic measurement setup, virtual gate matrices, single-particle spectrum and lever-arm extraction methods, limitations of the Fock–Darwin model with comparison to the Luttinger–Kohn Hamiltonian, analysis of energy-level discontinuities, tunnel-rate extraction via pulsed excited-state spectroscopy (PESS), comparison of Zeeman and orbital energy scales within the constant-interaction model, dependence of addition spectra with barrier voltage, pulsed excited-state spectroscopy of quantum dot 2, and magnetic-field-orientation dependence of CBAS and PESS measurements (PDF)
