# Supplementary material for: Disentangling Orbital and Confinement Contributions to g‑Factor in Ge/SiGe Hole Quantum Dots
Source: Nano Lett. 2026 Jun 17;26(25):8117–23. doi: 10.1021/acs.nanolett.6c00999 (PMC13329982; doi:10.1021/acs.nanolett.6c00999)
Supplement: Supplementary file 1 [file nl6c00999_si_001.pdf]

Supplementary information for: Disentangling orbital and confinement contributions to g-factor in Ge/SiGe hole quantum dots

L. Sommer,[1,*] I. Seidler,[1] F. J. Schupp,[1] S. Paredes,[1] N. W. Hendrickx,[1] L. Massai,[1] K. Tsoukalas,[1] A. Orekhov,[1] E. G. Kelly,[1] S. W. Bedell,[2] G. Salis,[1] M. Mergenthaler,[1] P. Harvey-Collard,[1] A. Fuhrer,[1] and T. Ihn[3]

[1] *IBM Research Europe – Zürich, Säumerstrasse 4, 8803 Rüschlikon, Switzerland*
[2] *IBM Quantum, T. J. Watson Research Center, 1101 Kitchawan Road, Yorktown Heights, New York 10598, USA*
[3] *Solid State Physics Laboratory, ETH Zürich, 8093 Zürich, Switzerland*

S1. METHODS

Device fabrication

The devices are based on a Ge/SiGe heterostructure with a 20 nm-thick strained Ge quantum well located 48 nm below the wafer surface. The heterostructure, with a $Si_{0.2}Ge_{0.8}$ barrier composition, is grown using an industrial reduced-pressure chemical vapor deposition (RP-CVD) process. Ohmic contacts to the quantum well are formed by platinum diffusion at 300 °C, resulting in low-resistance Pt-silicide contacts. The gate layout is fabricated in two layers. In the first layer, electrostatic gates are defined via electron-beam lithography and lift-off of a 20 nm Ti/Pd metal stack. A 7 nm-thick SiO_2 dielectric, deposited by plasma-enhanced atomic layer deposition (PE-ALD), electrically isolates the gate layers. This study includes measurements from two devices fabricated in separate runs on the same wafer, demonstrating reproducibility across fabrication batches.

Experimental setup

All measurements were performed in a Bluefors LD400 dilution refrigerator with a base temperature of $T_{mxc} \approx$ 15 mK. The device, mounted on a QDevil QBoard circuit board, was controlled via static gate voltages applied using a QDevil QDAC. DC lines were filtered at the mixing chamber stage using QDevil QFilters, guided through low-noise looms. Sensor conductance was measured using two Basel Precision Instruments (BasPI) SP983c IV converters (gain: 10^9, output low-pass filter: 300 Hz), with a source-drain bias of V_{SD} =200 µV for device 1 and 500 µV for device 2. The output was recorded using a Keysight 34461A digital multimeter. Magnetospectroscopy was conducted using an American Magnetics three-axis vector magnet, capable of applying fields up to (1, 1, 6) T in the (x, y, z) directions, with high-stability current sources on all axes. The device was centered with respect to the z-axis solenoid. Minor angular misalignments in tilted field measurements can be caused by imperfect planar mounting. Magnetic hysteresis effects, typically a few mT, were negligible in our field range. Pulsed excited-state spectroscopy was performed on a second, nominally identical device in a separate Bluefors LD400 fridge (base temperature ≈ 13 mK) using the same electronics. For pulsing, bias tees with a 1.1 ms time constant were employed. The high-frequency lines had an attenuation of 22 dB.

Virtual gate matrices

To mitigate capacitive crosstalk between the different electrostatic gates and the quantum dots, the following virtual gates [1] are used for the device 1:

$$\begin{pmatrix} V_{\mathrm{P1}} \\ V_{\mathrm{P2}} \\ V_{\mathrm{P3}} \\ V_{\mathrm{B12}} \\ V_{\mathrm{CP2}} \\ V_{\mathrm{RB2}} \end{pmatrix} = \begin{pmatrix} 1 & -0.220 & -0.092 & 0 & 0 & 0 \\ 0 & 1 & 0 & 0 & 0 & 0 \\ -0.105 & -0.239 & 1 & -0.381 & 0 & 0 \\ 0 & 0 & 0 & 1 & 0 & 0 \\ -0.4 & 0 & 0 & 0 & 1 & 0 \\ -0.4 & 0 & 0 & 0 & 0 & 1 \end{pmatrix} \begin{pmatrix} V_{\overline{\mathrm{P1}}} \\ V_{\overline{\mathrm{P2}}} \\ V_{\overline{\mathrm{P3}}} \\ V_{\overline{\mathrm{B12}}} \\ V_{\overline{\mathrm{CP2}}} \\ V_{\overline{\mathrm{RB2}}} \end{pmatrix}$$

V_{Gi} are the real and $V_{\overline{\mathrm{Gi}}}$ the virtual gate voltage, which leaves the chemical potentials of the nearby quantum dots unchanged.

For the device 2 the virtual gatematrix is:

$$\begin{pmatrix} V_{\mathrm{P1}} \\ V_{\mathrm{P2}} \\ V_{\mathrm{P3}} \\ V_{\mathrm{B12}} \\ V_{\mathrm{B23}} \\ V_{\mathrm{B41}} \\ V_{\mathrm{RB1}} \\ V_{\mathrm{RB2}} \end{pmatrix} = \begin{pmatrix} 1 & -0.239 & 0 & 0 & 0 & 0 & 0 & 0 \\ -0.345 & 1 & 0 & 0 & 0 & 0 & 0 & 0 \\ -0.14 & -0.19 & 1 & -0.3 & -0.66 & -0.47 & -0.1 & -0.055 \\ 0 & 0 & 0 & 1 & 0 & 0 & 0 & 0 \\ 0 & 0 & 0 & 0 & 1 & 0 & 0 & 0 \\ 0 & 0 & 0 & 0 & 0 & 1 & 0 & 0 \\ 0 & 0 & 0 & 0 & 0 & 0 & 1 & 0 \\ 0 & 0 & 0 & 0 & 0 & 0 & 0 & 1 \end{pmatrix} \begin{pmatrix} V_{\overline{\mathrm{P1}}} \\ V_{\overline{\mathrm{P2}}} \\ V_{\overline{\mathrm{P3}}} \\ V_{\overline{\mathrm{B12}}} \\ V_{\overline{\mathrm{B23}}} \\ V_{\overline{\mathrm{B41}}} \\ V_{\overline{\mathrm{RB1}}} \\ V_{\overline{\mathrm{RB2}}} \end{pmatrix}$$

S2. SINGLE-PARTICLE SPECTRUM EXTRACTION

The magnetospectroscopy data is acquired by stepping the magnetic field from −3 T to 3 T, while sweeping $V_{\overline{\mathrm{P1}}}$ from positive to negative and back. The derivative $\mathrm{d}I_{\mathrm{det}}/\mathrm{d}V_{\overline{\mathrm{P1}}}$ is normalized to its maximum value, and peak

* Correspondence to: lisa.sommer@ibm.com

positions are extracted. To suppress effects from charge rearrangements, drift, and other slow variations, the differences of two adjacent peaks are calculated. Both forward and backward sweeps are included to capture potential hysteretic behavior, and the final dataset represents the mean of the two. Error bars shown in Fig. 1e correspond to the standard deviation of this mean. Since the energy differences are symmetric with respect to magnetic field, the data are plotted as a function of the absolute value of $B_\perp$, resulting in two data points per magnetic field value. While for CBAS the sensor is retuned for each $B_\perp$ value, this is not done for PESS since it remained sufficient sensitivity throughout the magnetic field sweep.

S3. LEVER ARM EXTRACTION

The lever arms of the virtual gates $V_{\overline{\mathrm{P1}}}$ and $V_{\overline{\mathrm{P2}}}$ were extracted using temperature broadening of Coulomb peaks as a function of the fridge temperature. To this end, Coulomb blockade transitions were measured at $B_\perp = 0\,\mathrm{T}$ in both sweep directions and repeated multiple times (Fig. S1). Higher occupancies have higher uncertainty due to reduced measurement sensitivity. Each transition was fitted using a Fermi-Dirac distribution after background subtraction, normalization, and voltage offset correction to center the transition around zero:

$$f(V) = \frac{A}{e^{\frac{\alpha(V-V_0)}{k_B T_\mathrm{e}}} + 1} \tag{S1}$$

Here, the amplitude A and the voltage offset V_0 are known parameters, which were allowed to vary during the fitting, and the primary fitting parameter is the ratio $\frac{T_\mathrm{e}}{\alpha}$, where T_e is the base electron temperature and α is the lever arm. Fits were performed across multiple datasets, and only those with $R^2 > 0.9$ were retained for further analysis. The extracted T_e/α values were plotted against the stabilized fridge temperature T_fridge (Fig. S2a,c,e and Fig. S3a,c). Temperature readings were taken after a 15 min stabilization period. The heater was mounted close to the device, while the temperature sensor was positioned on the opposite side, potentially introducing a thermal gradient. However, both components were thermally contacted to the probe's metal body, ensuring good thermal contact and minimizing systematic errors in T_fridge.

At low temperatures, the broadening remains constant up to approximately 200 mK, indicating a base electron temperature T_e. Beyond this point, the broadening increases linearly with T_fridge. A linear fit with zero intercept yields the lever arm α as the slope. Higher temperatures lead to increased data scatter, reducing fit accuracy. The extracted lever arms for different hole numbers and for the second device (including out-of-plane magnetic field data) are shown in Fig. S2b,d,f and Fig. S3b,d. Uncertainties represent the standard deviation of the fit and are in some cases smaller than the marker size. However, the spread in T_e/α resulting in a minimum uncertainty of 10 %, which propagates into the lever arm estimation.

Across all datasets, the lever arm values consistently converge around $\alpha \approx 0.1$.

To validate the lever arm extraction, we employed an independent method based on transport measurements. In this approach, a finite bias was applied across the double quantum dot in addition to the sensor bias. Bias triangles were recorded at the $(1,0)-(0,1)$ interdot transition. The lever arm was then determined by dividing the applied bias voltage by the extent of the bias triangles along the gate voltage axis, as indicated by the dashed lines in Fig. S4. For device 1, this analysis yielded lever arms of $\alpha_{V_{\overline{\mathrm{P1}}}} = 0.113$ and $\alpha_{V_{\overline{\mathrm{P2}}}} = 0.103$, while for device 2, we obtained $\alpha_{V_{\overline{\mathrm{P1}}}} = 0.100$ and $\alpha_{V_{\overline{\mathrm{P2}}}} = 0.077$. Based on the consistency of these values, we adopt a representative lever arm of 0.1 for both virtual plunger gates ($V_{\overline{\mathrm{P1}}}$ and $V_{\overline{\mathrm{P2}}}$) across both devices throughout this work.

S4. LIMITATIONS OF THE FOCK-DARWIN MODEL

For the CBAS within the isotropic Fock-Darwin model, energy spectrum is given by [2]:

$$E_{n,l} = \hbar\Omega(2n + |l| + 1) - \frac{1}{2}\hbar\omega_c l \pm \frac{1}{2}g_N^*\mu_B B$$

where $\Omega = (\omega_N^2 + (\omega_c/2)^2)^{(1/2)}$, ω_N characterizes the confinement potential of the dot with N holes, g_N^* is the effective g-factor for hole number N and $\omega_c = eB/m^*$. To analyze the influence of the orbital state on the slope of the energy-level difference, we calculate it explicitly for small magnetic fields ($B < 0.2\,\mathrm{T}$), where higher-order terms in B^4 can be neglected. As an example, we consider the first two levels, $E_{0,0}$ and $E_{0,1}$, both with spin $+1/2$. The difference is given by:

$$E_{0,1} - E_{0,0} = \hbar\underbrace{(\omega_1 - \omega_0)}_{<0} + \frac{1}{2}\left(\frac{eB}{2m}\right)^2 \underbrace{\left(\frac{2}{\omega_1} - \frac{1}{\omega_0}\right)}_{>0} + \frac{\hbar\omega_c}{2} \mp \frac{1}{2}\mu_B B(g_1^* - g_0^*)$$

This analysis reveals that the change in the confinement potential by hole number N increase introduces a quadratic dependence on the magnetic field. While this effect may not be directly visible in the raw data, it becomes apparent in the residuals of the linear fits, as shown in Fig. S6 and Fig. S5. Notably, a clear deviation from linearity is observed only in the extraction of g^{S-T_-}, indicating that the inclusion of different orbital states seems to introduce an additional B^2 effect. For the other extracted g-factors, the residuals remain featureless, suggesting that changes in the confinement potential are negligible in those cases even in CBAS $g^{\epsilon_2-\epsilon_1}$ and $g^{\epsilon_4-\epsilon_3}$, where it should be most evident Fig. S5. This shows the limitations of the Fock-Darwin model.

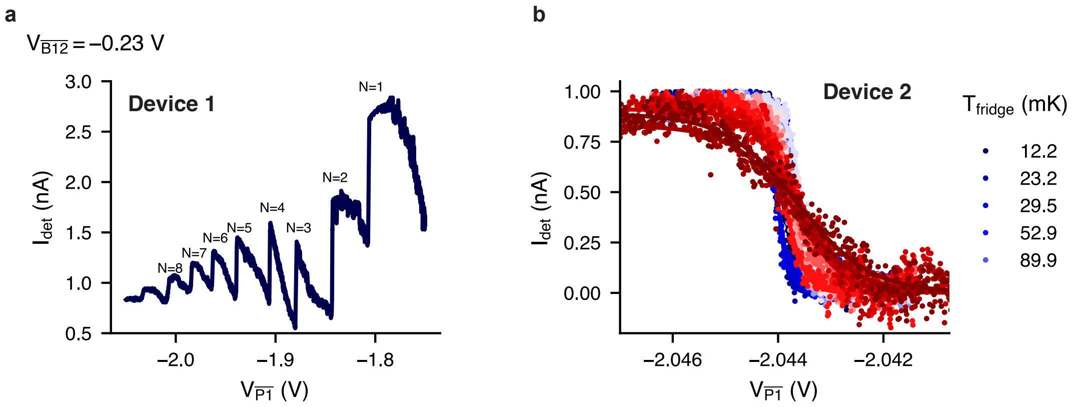

Figure S1. **Extraction of the lever arm from temperature-broadened transitions.** **(a)** Example dataset showing eight charge transitions measured via the sensor current I_{SD} for device 1 at $V_{\overline{B12}}$ is $-0.23\,$V. **(b)** Zoom-in of the first transition, measured on device 2. The sensor current is normalized, and the transition is fit using a Fermi–Dirac distribution at various temperatures, illustrating the thermal broadening.

Assuming an anisotropic Fock-Darwin model for including an ellipsoidal dot the energy spectrum is given by [3]:

$$E_{n_x,n_y} = (n_x + \frac{1}{2})\hbar\omega_1 + (n_y + \frac{1}{2})\hbar\omega_2 \pm \frac{1}{2} g_N^* \mu_B B$$

where $\omega_1 = \alpha_1\beta_1/m_e$ and $\omega_2 = \alpha_2\beta_2/m_e$ with

$$\alpha_1^2 = \frac{\Omega_1^2 + 3\Omega_2^2 + \Omega_3^2}{2(\Omega_1^2 + \Omega_2^2)}, \beta_1^2 = \frac{1}{4}(3\Omega_1^2 + \Omega_2^2 + \Omega_3^2),$$

$$\alpha_2^2 = \frac{3\Omega_1^2 + \Omega_2^2 - \Omega_3^2}{2(\Omega_1^2 + \Omega_2^2)}, \beta_2^2 = \frac{1}{4}(\Omega_1^2 + 3\Omega_2^2 - \Omega_3^2),$$

$$\Omega_{1,2}^2 = m_e^2(\omega_{x,y}^2 + \frac{1}{4}\omega_c^2)$$

$$\Omega_3^2 = [(\Omega_1^2 - \Omega_2^2)^2 + 2m_e^2\omega_c^2(\Omega_1^2 + \Omega_2^2)]^{1/2}$$

$$\omega_c = eB/m_e c$$

Using the same analysis as before and assuming that the ellipsoidal dot elongates with increasing hole number, resulting in $\omega_{1,0} > \omega_{1,1}$ and $\omega_{2,0} > \omega_{2,1}$, the calculation (not shown here due to its length) reveals a B^2 dependence additionally to $\mp\frac{1}{2}\mu_B B(g_1^* - g_0^*)$.

To account for a linear variation in the effective g-factor, which is visible in the residuals of $\uparrow_{o1} - \downarrow_{o2}$, it is necessary to go beyond the simple Zeeman Hamiltonian and consider the full Luttinger–Kohn (LK) Hamiltonian. In particular, the off-diagonal elements of the Zeeman Hamiltonian for a perpendicular magnetic field $\vec{B} = B\hat{z}$ are given by:

$$H_Z = 2\mu_B \left(\kappa B J_z + qB - J_z^3\right),$$

where κ and q are the isotropic and cubic Zeeman parameters, respectively. For states with angular momentum difference $\Delta l = 1$, such as the heavy-hole (HH) state $|HH+\rangle = |3/2, +3/2\rangle$ and the light-hole (LH) state $|LH+\rangle = |3/2, +1/2\rangle$, the spherical approximation does not allow coupling via orbital angular momentum. However, the cubic Zeeman term introduces non-zero matrix elements such as:

$$\langle 3/2, +1/2|\, J_z^3 \,|3/2, +3/2\rangle \neq 0,$$

indicating that heavy-hole-light-hole mixing can occur through this term.

To capture this mixing more accurately, we consider the full Luttinger–Kohn Hamiltonian:

$$H = \frac{\hbar^2}{2m_0}\left[\left(\gamma_1 + \frac{5}{2}\gamma_2\right)k^2 - 2\gamma_2(\vec{k}\cdot\vec{J})^2\right],$$

where γ_1 and γ_2 are Luttinger parameters, and $\vec{J}$ is the total angular momentum operator for $j = 3/2$. Applying the Peierls substitution $\vec{k} \to \vec{k} + \frac{e}{\hbar}\vec{A}$ with the symmetric gauge $\vec{A} = \frac{B}{2}(-y, x, 0)$, we obtain:

$$k_x \to k_x - \frac{eB_z}{2\hbar}y, \quad k_y \to k_y + \frac{eB_z}{2\hbar}x.$$

The heavy-hole-light-hole mixing (HH-LH) arises from the off-diagonal terms in the LK Hamiltonian, which are proportional to:

$$H_{HH-LH} \propto k_+^2 J_-^2 + k_-^2 J_+^2,$$

with $k_\pm = k_x \pm ik_y$ and $J_\pm = J_x \pm iJ_y$. Substituting the magnetic field dependence into k_+, we find:

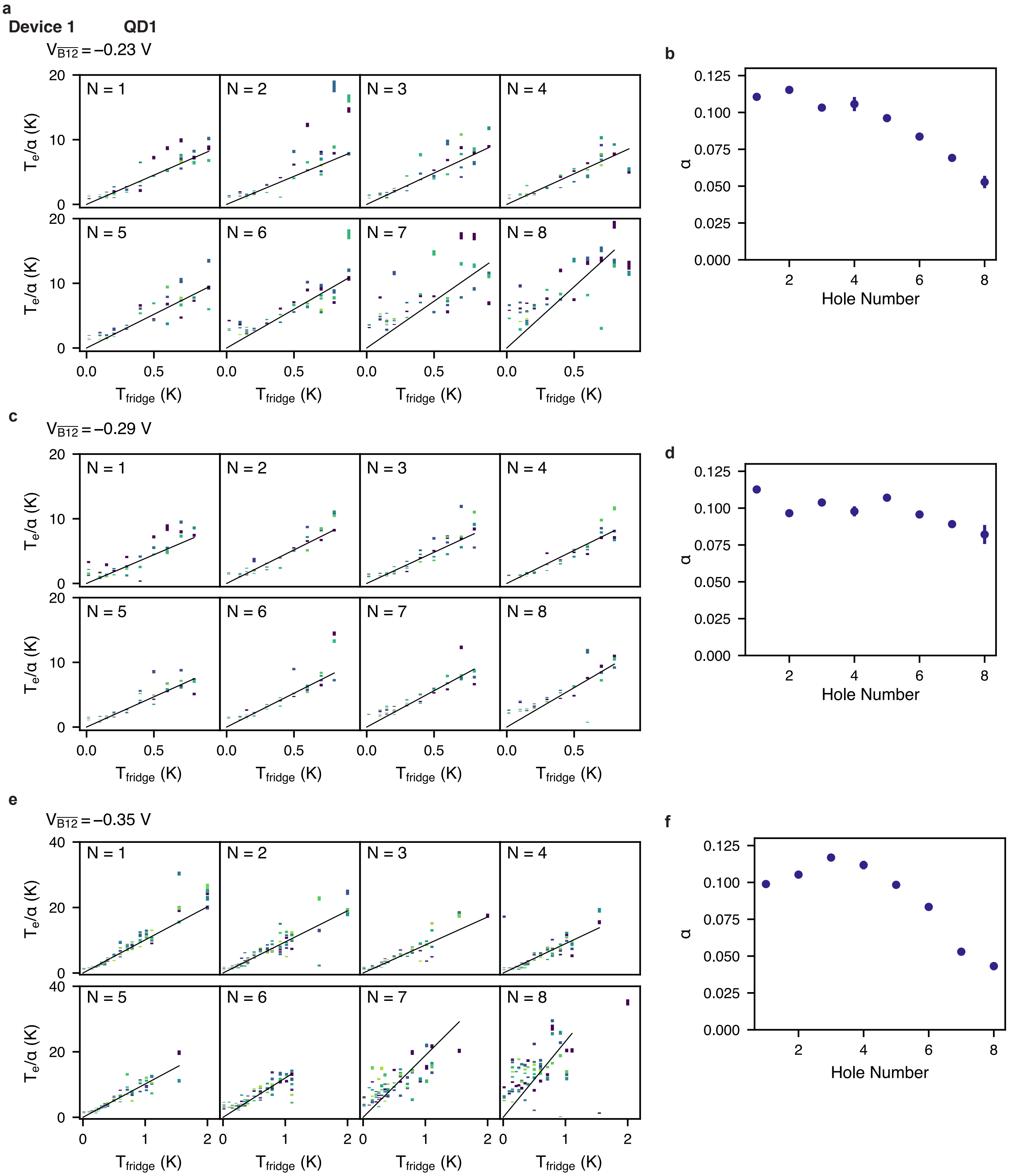

Figure S2. **Extraction of the lever arm from temperature-broadened transitions for device 1. (a, c, e)** Width of the Fermi–Dirac distribution extracted from the data in Fig. S1, plotted as a function of fridge temperature for the Nth transition, and fitted for different values of $V_{\overline{\mathrm{B12}}}$. **(b, d, f)** Resulting lever arms plotted as a function of hole number N for the corresponding $V_{\overline{\mathrm{B12}}}$ values.

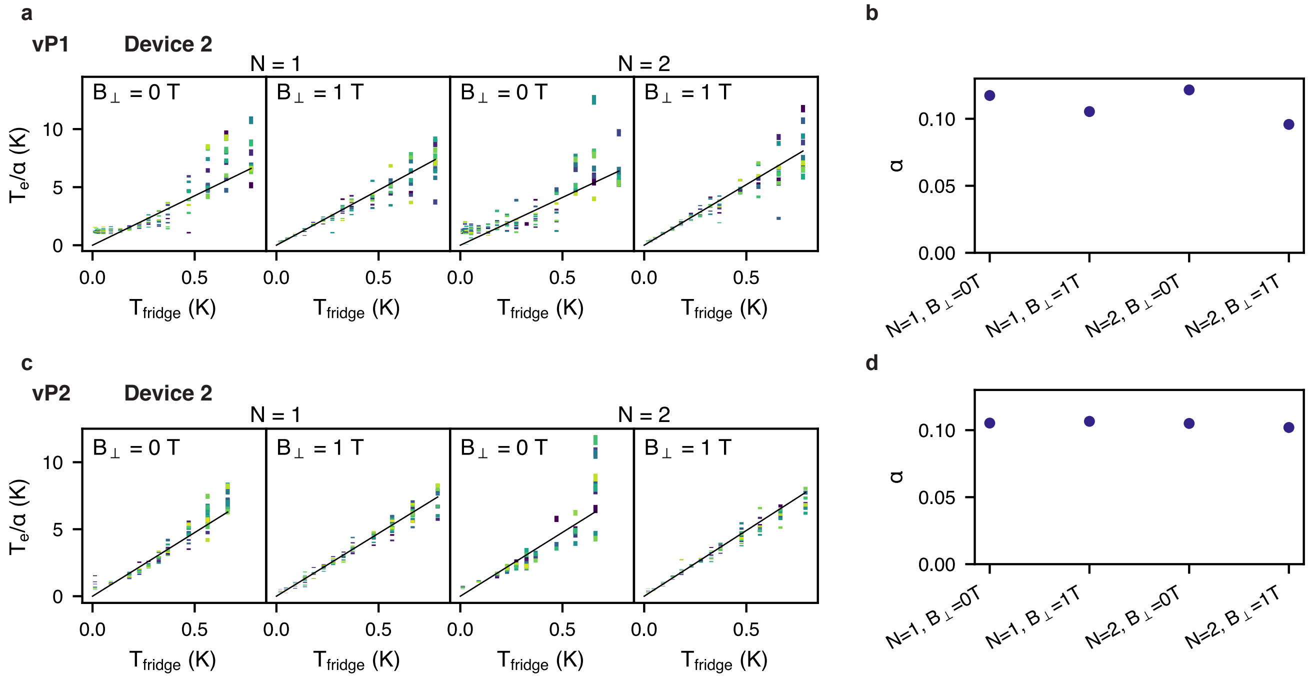

Figure S3. **Extraction of the lever arm from temperature-broadened transitions for device 2. (a, c)** Width of the Fermi–Dirac distribution extracted from the data in Fig. S1, plotted as a function of fridge temperature for the Nth transition, and fitted for different out-of-plane magnetic fields $B_\perp$. **(b, d)** Resulting lever arms plotted as a function of hole number N and magnetic field $B_\perp$.

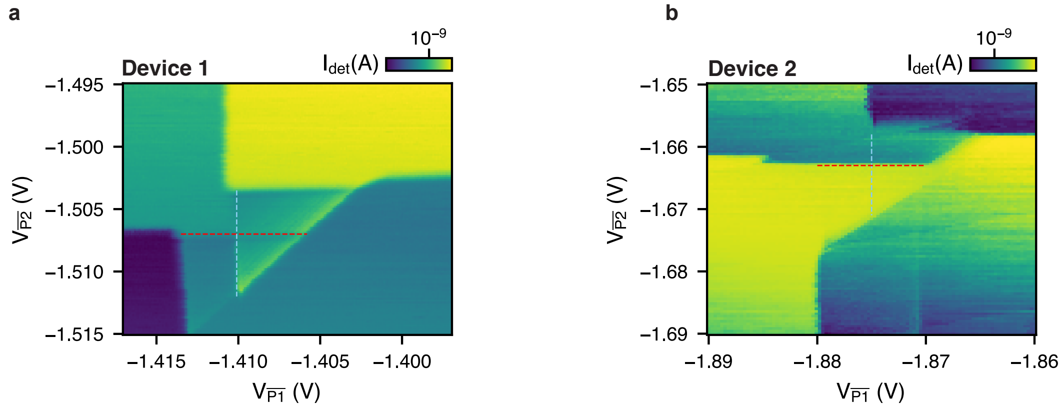

Figure S4. **Extraction of the lever arm from bias triangles.** Bias triangles are measured by applying a source–drain bias of 1 mV to the double quantum dot. Dashed lines indicate the voltage span used for lever arm extraction. **(a)** Measurement of device 1. **(b)** Measurement of device 2.

$$k_+ = k_+^{(0)} + \frac{eB_z}{2\hbar}(-y + ix),$$

so that k_+^2 contains terms linear in B. Therefore, the heavy-hole-light-hole mixing leads to a linear modification of the effective g-factor.

S5. DISCONTINUITY

To investigate the influence of orbital effects, hole-hole interactions, and confinement on the energy spectrum, we compare the addition spectrum (CBAS) with the single-particle excitation spectrum (PESS), both extracted from the same magnetospectroscopy dataset. The CBAS, shown in Fig. 2a, is derived from the ground states for different hole numbers in Fig. 2d using the previously described method. For transitions $N = 1 \leftrightarrow 2$

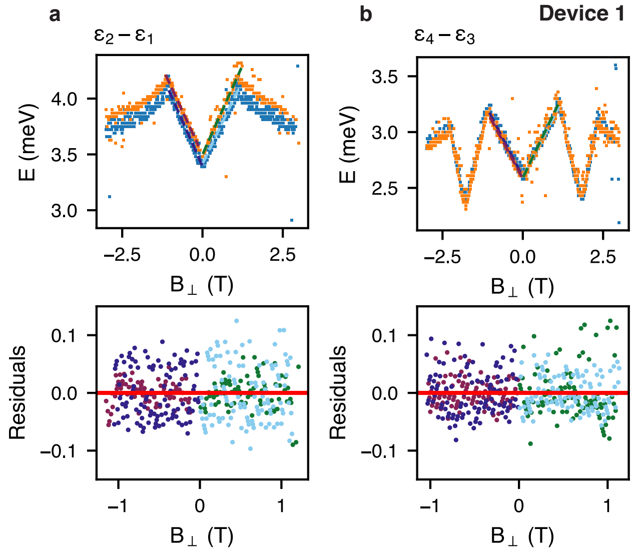

Figure S5. **Residual analysis from linear fits to CBAS data of device 1** Residuals are extracted from linear fits to the difference of levels within the CBAS measurements shown in Fig. 1e. Forward and backward magnetic field sweeps and voltage sweeps are analyzed separately to better resolve potential trends. **(a)** g-factor extraction for the ground states of $N = 1, 2$, as obtained from CBAS measurements ($g^{\epsilon_2-\epsilon_1}$). The residuals show no significant trend. **(b)** g-factor extraction for the ground states of $N = 3, 4$, as obtained from CBAS measurements ($g^{\epsilon_4-\epsilon_3}$). The residuals show no significant trend.

and $N = 2 \leftrightarrow 3$, the CBAS ground-state energies exhibit a discontinuity near $B_\perp = 1.49\,\mathrm{T}$, indicated by a yellow dashed line. This feature is also visible when overlaying the data from Fig. 1e with an anisotropic Fock-Darwin model as described in Ref. [3], shown in Fig. S7. While the model captures the general trend of the energy levels, deviations occur at level crossings and for higher excited states.

The observed discontinuity likely originates from changes in the confinement potential as hole occupancy increases, modifying orbital energies and shifting magnetic-field crossings. To gain insight into the origin of the observed discontinuities, we simulate the system using an anisotropic Fock–Darwin model, following the approach of Madhav and Chakraborty [3]. By varying the g-factors for the different Zeeman-split pairs (see Fig. S8a), as measured for device 1 (see Fig. 1e), a discontinuity emerges at the first level crossing. This arises because the two crossing states have different g-factors and therefore also different ϵ_1, while the offset is always subtracted starting at 0 meV. This requires adapting the chemical potential description from Ref. [4] to account for level-specific energies for each hole N:

$$\mu(1) = E_{0,0}(N = 0) = \epsilon_1$$
$$\mu(2) = E_c + E_{0,0}(N = 1)$$

In contrast, the next higher crossing does not exhibit a discontinuity, as both involved states share the same g-factor. However, as shown in Fig. Fig. 1e and Fig. S7, discontinuities are also present at higher crossings (for each level, the first level is adapted to the parameters). These can be explained by changes in the anisotropy of the confinement potential, as illustrated in Fig. S8b. Due to the number of free parameters for each energy level $(g(N), \omega_x(N), \omega_y(N), m^*(N))$, the risk of overfitting is high and offers limited physical insights. Additionally, spin–orbit interactions may contribute to such discontinuities, as discussed by Bulaev and Loss [5]. These findings highlight the sensitivity of the energy spectrum to both orbital configuration and spin-dependent interactions, emphasizing the challenge for simple models. Therefore this shows the need for detailed modeling to interpret experimental observations.

To investigate the discontinuity observed in the addition spectrum further, we examine the PESS data near the singlet–triplet crossover (Fig. S9a). Here, the discontinuity is not observed in the PESS data but appears clearly in the CBAS measurements, where each energy level corresponds to the addition of a hole. This contrast highlights the role of many-body interactions and confinement changes upon hole addition, which are more pronounced in the addition spectrum. Furthermore, no anticrossing is visible in the PESS data. To extract an upper bound on the Rashba spin–orbit coupling gap at the degeneracy point [5, 6], we determine the full width at half maximum (FWHM) of the transition line to be 52.7(181) µeV.

In a slightly modified configuration of tunnel rates, the singlet branch beyond the crossing becomes faintly visible in Fig. S9b. Here, the crossing is measured by tuning both plunger gates, demonstrating that the g-factor voltage tunability is sufficient to traverse the crossover. This suggests that hole–hole interactions and modifications to the confinement potential upon hole addition significantly influence the energy spectrum—effects that are less pronounced in the PESS data.

S6. TUNNEL RATE MEASUREMENT FOR PESS

To measure the tunnel rates of different ground and excited states, we sweep the AC pulse frequency while monitoring the response as a function of DC gate voltage (Fig. S10). Ground and excited states appear as lines in the data, which indicate tunneling events between the quantum dot and the reservoir. If the tunnel rates were lower than the pulse frequency (repetition rate), these lines would disappear. Since the lines remain visible down to the lowest measurable frequency (1 kHz), we conclude that the tunnel rate is above the detection limit but still sufficient to observe transitions.

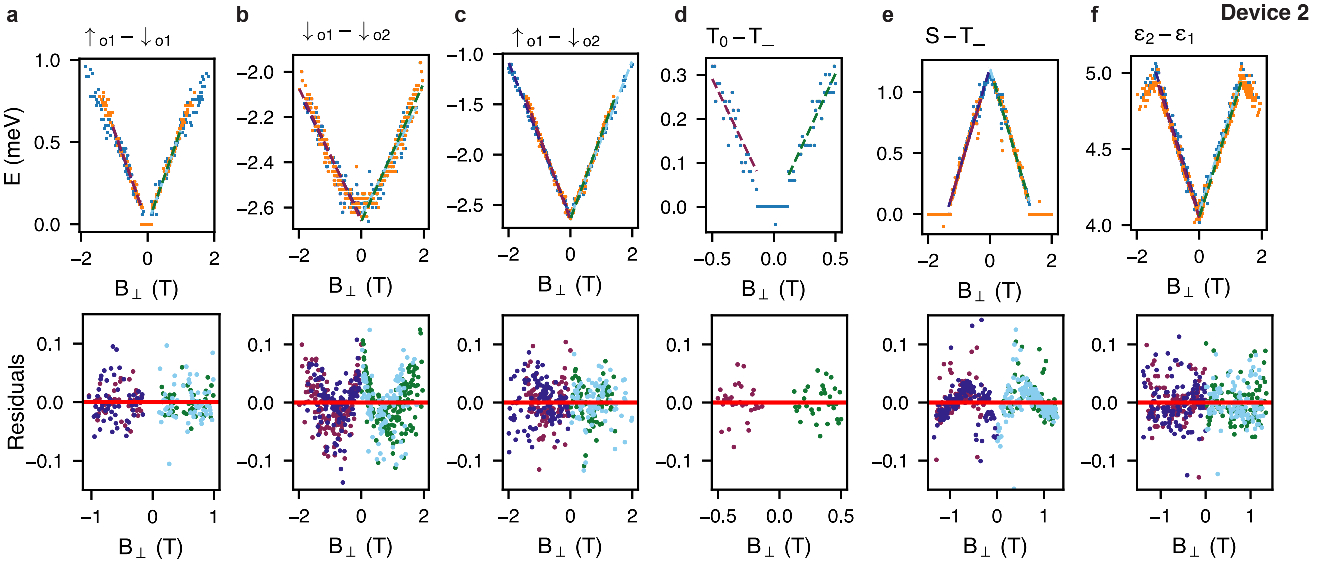

Figure S6. **Residual analysis from linear fits to PESS data of device 2** Residuals are extracted from linear fits to the difference of levels within the PESS measurements shown in Fig. 2d. Forward and backward magnetic field sweeps and voltage sweeps are analyzed separately to better resolve potential trends. **(a)** Extraction of the pure spin g-factor for the $N = 1$ ground state ($g^{\uparrow_{o1}-\downarrow_{o1}}$). The residuals show no significant trend. **(b)** linear fit extraction for $N = 1$ including the first excited orbital state($\downarrow_{o1} - \downarrow_{o2}$). The residuals show a systematic deviation, suggesting an orbital contribution. **(c)** g-factor extraction for $N = 1$ including the first excited orbital state($g^{\uparrow_{o1}-\downarrow_{o2}}$). The residuals show a large spread, but no systematic deviation. **(d)** Pure spin g-factor extraction for the $N = 2$ transition ($g^{T_0-T_-}$). The residuals show no significant trend. **(e)** g-factor extraction for $N = 2$ including the lower orbital state (g^{S-T_-}). The residuals exhibit a systematic deviation, suggesting an additional orbital contribution that introduces a B^2 dependence. **(f)** g-factor extraction for the ground states of $N = 1, 2$, as obtained from CBAS measurements ($g^{\epsilon_2-\epsilon_1}$). The residuals show no significant trend.

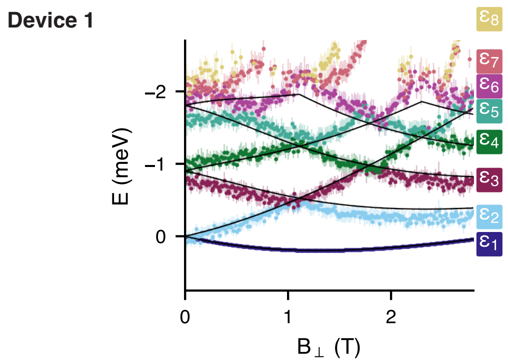

Figure S7. **Fock-Darwin simulation of data from Fig. 1e** using $m^* = 0.09m_e$, $\hbar\omega_x = 0.9\,\text{meV}$, $\hbar\omega_y = 2\,\text{meV}$ and $g = 11.12$

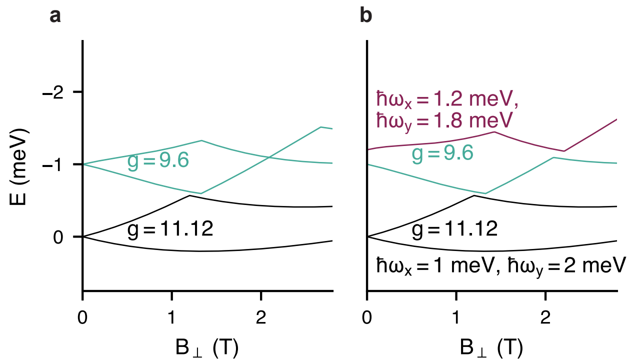

Figure S8. **Fock-Darwin simulation of quantum dot with decreased g-factor and increased anisotropy with hole number. (a)** Simulated Zeeman-split energy levels with varying g-factors, indicated by color. The confinement potential is given by $\hbar\omega_x = 1\,\text{meV}$, $\hbar\omega_y = 2\,\text{meV}$ and the effective mass is $m^* = 0.09$ **(b)** The same g-factors as in panel a are used, but the confinement potential is modified for the fourth orbital level to reflect variation of the anisotropy. The first three levels retain the original confinement parameters.

S7. COMPARISON OF ENERGY SCALES

To understand the impact of orbital in comparison to the spin, we calculate the energy scales within the constant interaction model. Zeeman energy, E_Z, is given by:

$$E_Z = g^* \mu_B B = g^* \frac{e\hbar B}{2m_e}$$

where m_e is the electron mass. The orbital energy, E_{orb} scale is given by:

$$E_{\text{orb}} = \hbar\omega_c = \frac{\hbar e B}{m^*}$$

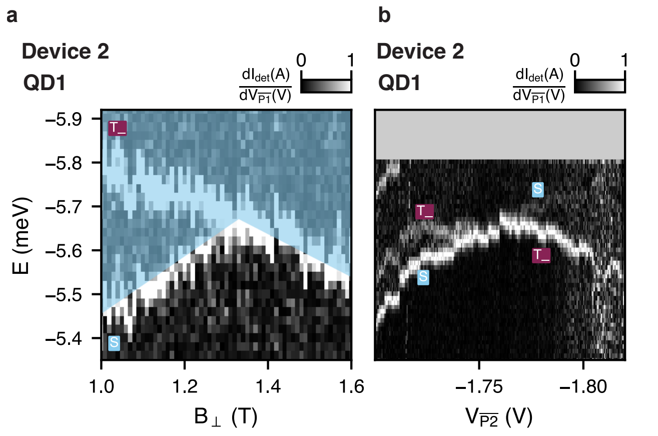

Figure S9. **Zoom-in of Fig. 2d at the singlet–triplet crossing. (a)**, Measured as a function of magnetic field. **(b)**, Measured via voltage-tunable g-factor by varying the plunger gate of the second, which is the unoccupied QD2 at $N = 0$.

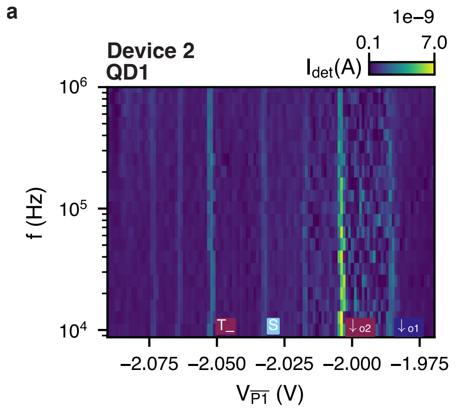

Figure S10. **Tunnel rate extraction from AC pulse spectroscopy with $B_\perp$ =2.5 T, $\delta V_{\overline{\mathrm{P1}}}$ =0.2 V, $V_{\overline{\mathrm{P2}}} = -1.6$ V.** AC pulse frequency is swept against the DC gate voltage applied to QD1 of device 2 to probe the tunnel rate.

where $m^* = 0.09 \pm 0.002 m_e$ [7] is the effective mass. Using $g^* = 10$, the proportion is

$$\frac{E_Z}{E_{\mathrm{orb}}} = 0.45$$

Therefore, the energy scales are comparable and it is a strong spin-orbit coupled system.

S8. ADDITION SPECTRA IN DEPENDENCE OF $V_{\overline{B12}}$

In Fig. S11, the charge stability diagrams for the double quantum dot system are shown for decreasing values of $V_{\overline{B12}}$. As $V_{\overline{B12}}$ becomes more negative, the tunnel coupling between the two quantum dots increases, as evidenced by the broadening at the triple points.

To extract the ellipticity of QD1, we analyze the energy difference $\epsilon_5 - \epsilon_4$ at $B = 0$ T for decreasing values of $V_{\overline{B12}}$. The results are plotted in Fig. 3, where, for $N = 4$, the single-particle energy is smaller at $V_{\overline{B12}} = -0.35$ V compared to higher voltages. This indicates that the quantum dot becomes less elliptical as $V_{\overline{B12}}$ is made more negative, with the confinement potential approaching a less ellipsoidal confinement potential shape at $V_{\overline{B12}} = -0.35$ V relative to $V_{\overline{B12}} = -0.23$ V. A discontinuity is visible at level crossings in all spectra. The voltage tunability of the g-factor is directly evident from the crossing of ϵ_2 and ϵ_3, which occurs at different magnetic fields for different values of $V_{\overline{B12}}$. This observation indicates that, although the overall confinement potential shape of the quantum dot does not change drastically, the g-factor is significantly affected by the gate voltage.

S9. PULSED EXCITED-STATE SPECTROSCOPY OF DOT UNDER $V_{\overline{P2}}$

PESS measurements were also performed on the second quantum dot (QD2) in the double quantum dot (DQD) system of device 2. However, due to the complex voltage landscape near QD2—likely influenced by the proximity of the charge sensor, we were unable to optimize the tunnel couplings to resolve additional excited states beyond those shown in Fig. S14a. From the visible spin-split pair, we extract the differences of the levels at $B_\perp = 0$ T $\Delta\mathrm{orb}_{N_{0\leftrightarrow 1}} = 2.76$ meV, $\Delta\mathrm{orb}_{N_{1\leftrightarrow 2}} = 1.36$ meV. In comparison to QD1, the differences are larger by 7.81% for $\Delta\mathrm{orb}_{N_{0\leftrightarrow 1}}$ and 27.1% for $\Delta\mathrm{orb}_{N_{1\leftrightarrow 2}}$. This indicates within the Fock-Darwin model, that the confinement is tighter for QD2.

Furthermore, we investigate the g-factors of QD2, which are extracted from Fig. S14b and listed in table S0. Compared to QD1, the PESS $g^{\uparrow_{o1}-\downarrow_{o1}}$-factor is reduced, while the PESS $g^{\uparrow_{o1}-\downarrow_{o2}}$-factor and PESS g^{S-T_0}-factor are significantly enhanced. Additionally, using the CBAS method to extract the orbital energy difference yields $g^{\epsilon_2-\epsilon_1} = 13.53(18)$, which aligns more closely with measurements taken at more negative barrier voltages as seen in Fig. 3b. These observations suggest that, despite nominally identical gate geometries, the two dots exhibit distinct confinement potentials, likely due to local strain variations or differing proximities to the charge sensor, which result in a modified potential landscape.

Additionally, we shifted the wavefunction toward the interdot charge transition using the plunger gate $V_{\overline{\mathrm{P1}}}$, as shown in the bottom panel of Fig. S14c, to investigate the tunability of the pure-spin PESS g-factor. Near the transition, both $g^{\uparrow_{o1}-\downarrow_{o1}}$ and $g^{T_0-T_-}$ exhibit an apparent increase. A similar trend for $g^{T_0-T_-}$ in QD1 is observed in Fig. 3c, indicating that the g-factor changes continuously as the wavefunction shifts from one plunger gate to the other.

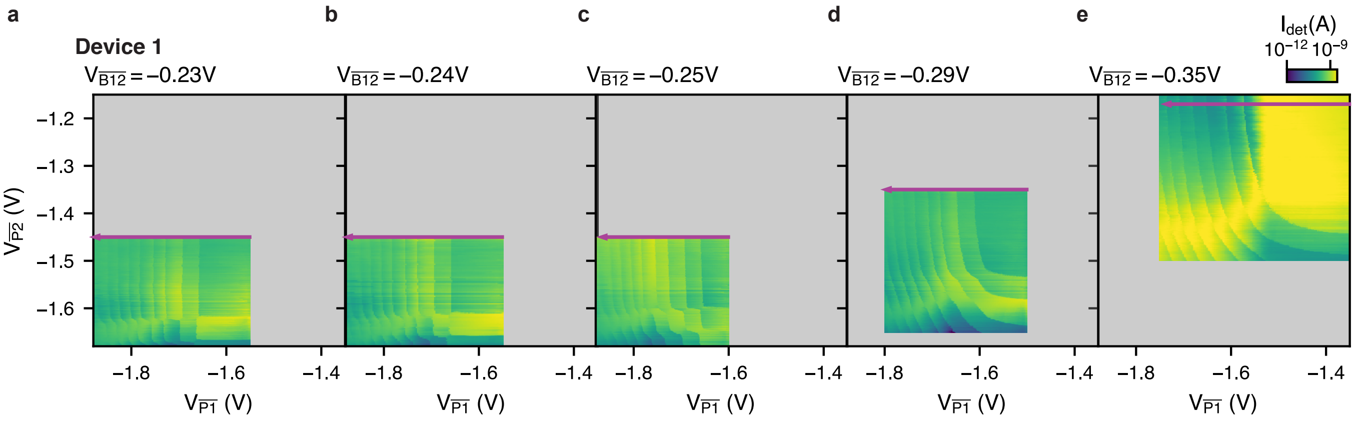

Figure S11. **Charge stability diagrams for different barrier voltages $V_{\overline{B12}}$ on device 1.** The purple arrows indicate the voltage configurations where the data for the addition spectrum was acquired. **(a)** $V_{\overline{B12}}$ =−0.23 V **(b)** $V_{\overline{B12}}$ =−0.24 V **(c)** $V_{\overline{B12}}$ =−0.25 V **(d)** $V_{\overline{B12}}$ =−0.29 V **(e)** $V_{\overline{B12}}$ =−0.35 V

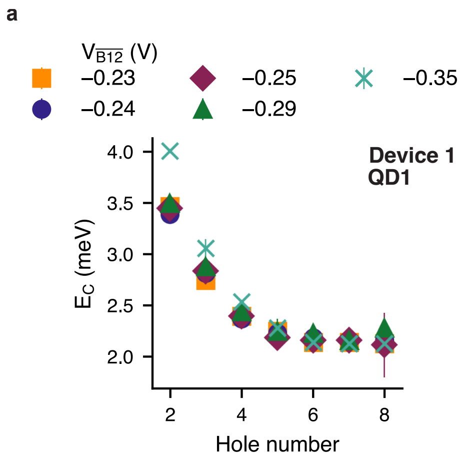

Figure S12. **(a)** Extracted charging energies (gray) for different barrier voltages $V_{\overline{B12}}$ as a function of total dot occupation. Error bars are smaller than the marker size.

Table S0. Extracted absolute g-factors with 3σ-error bars from CBAS and PESS for dot under $V_{\overline{P2}}$ of device 2, where N is the hole number. Additionally, the orbital number investigated in the analysis are indicated.

**Device 2 - QD2**				
	method		N	orbital
$g^{\epsilon_2-\epsilon_1}$	CBAS	13.53(54)	1,2	1
bare spin				
$g^{\uparrow_{o1}-\downarrow_{o1}}$	PESS	8.80(51)	1	1
spin & orbital				
$g^{\uparrow_{o1}-\downarrow_{o2}}$	PESS	14.72(66)	1	1,2
g^{S-T_-}	PESS	16.85(63)	2	1,2

S10. TILTED MAGNETIC FIELDS

Complementary to the magnetic field strength dependence, we also investigated the anisotropy of the g-factor, as reported in previous studies [8, 9]. For this, magnetospectroscopy measurements were performed as a function of the magnetic field angle θ, while maintaining a constant field magnitude of $|B| = 1$ T. Two in-plane field orientations were considered: $\phi = 0°$ and $\phi = 90°$. The analysis followed the procedure described in the main text. We assumed zero Zeeman splitting at $|B| = 0$ T, consistent with physical expectations. The g-factor was thus determined from the slope of the energy splitting.

The results, measured with CBAS for both in-plane angles overlapped within the experimental uncertainty of approximately 2°. The g-factor reaches its maximum when the magnetic field is oriented perpendicular to the quantum well plane ($\theta = 0°$ or $180°$), , indicating that any tilt of the g-tensor is below our detection threshold (see Fig. S15b). In contrast, the in-plane g-factor is significantly smaller, consistent with previous reports [8], and reaches values around 0.06, which is in the same range as previously reported values in literature [8, 10, 11]. These measurements were also performed at a fixed magnetic field magnitude of $|B| = 1$ T. A slight anisotropy is observed in the in-plane g-factor for the orbital splitting $g^{\epsilon_2-\epsilon_1}$. However, due to the relatively large experimental uncertainty and the possibility of misalignment between the device and the magnet's coordinate system, this anisotropy cannot be conclusively resolved. As such, more precise techniques, such as qubit spectroscopy, would be required to detect the in-plane anisotropy reported in earlier studies [8].

In addition, we investigated the single-particle excitation spectrum in a tilted magnetic field using the PESS method, as shown in Fig. S16a,b. The first excited state exhibits a strongly anisotropic g-factor, varying between approximately from 0 to 10 depending on the magnetic field orientation (Fig. S16c,d). However, no clear

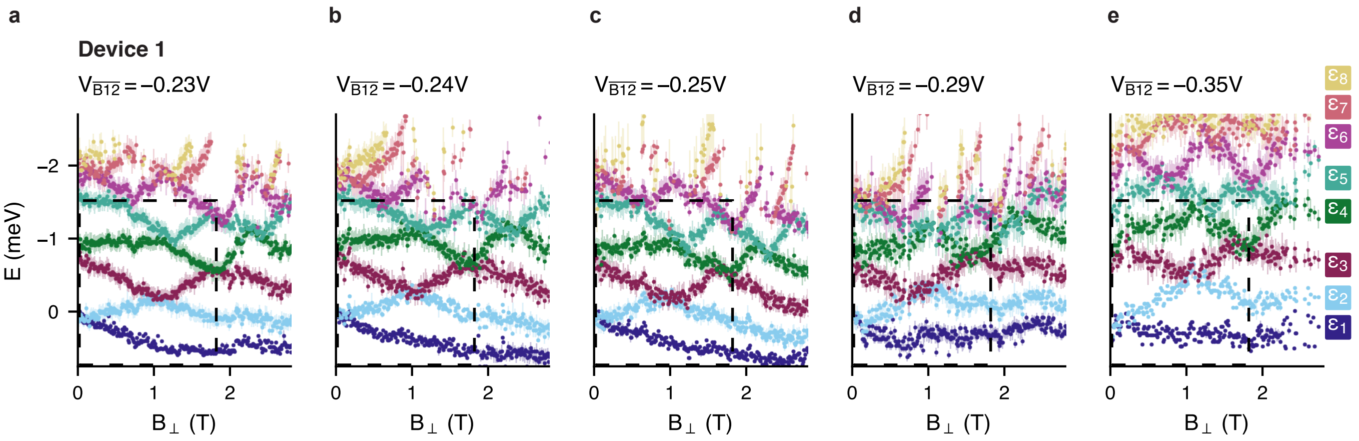

Figure S13. **Addition spectra for different barrier voltages $V_{\overline{B12}}$.** The data were obtained by subtracting the charging energy E_C (Fig. S12a) from the addition energies on device 1. The dashed square highlights the region used for comparison with Fig. 1e, Fig. 2a and between the panels. **(a)** $V_{\overline{B12}} = -0.23\,\mathrm{V}$ **(b)** $V_{\overline{B12}} = -0.24\,\mathrm{V}$ **(c)** $V_{\overline{B12}} = -0.25\,\mathrm{V}$ **(d)** $V_{\overline{B12}} = -0.29\,\mathrm{V}$ **(e)** $V_{\overline{B12}} = -0.35\,\mathrm{V}$

anisotropy is observed in the in-plane g-factor using this method (Fig. S16e), which may be attributed to a slight tilt of the device relative to the magnet coordinate system or a small residual out-of-plane magnetic field component. These findings are consistent with those obtained via the CBAS method, confirming that both approaches yield qualitatively similar conclusions. The quantitative differences arise from the nature of the two techniques: PESS probes single-hole excited states, whereas CBAS involves many-body effects, as discussed in the main text.

REFERENCES

[1] T. Hensgens, T. Fujita, L. Janssen, X. Li, C. J. Van Diepen, C. Reichl, W. Wegscheider, S. Das Sarma, and L. M. K. Vandersypen, "Quantum simulation of a fermi–hubbard model using a semiconductor quantum dot array," Nature **548**, 70 (2017).

[2] T. Ihn, *Semiconductor Nanostructures: Quantum states and electronic transport* (Oxford University Press, 2009).

[3] A. V. Madhav and T. Chakraborty, "Electronic properties of anisotropic quantum dots in a magnetic field," Phys. Rev. B **49**, 8163 (1994).

[4] L. P. Kouwenhoven, D. G. Austing, and S. Tarucha, "Few-electron quantum dots," Reports on Progress in Physics **64**, 701 (2001).

[5] D. V. Bulaev and D. Loss, "Spin relaxation and decoherence of holes in quantum dots," Phys. Rev. Lett. **95**, 076805 (2005).

[6] D. V. Bulaev and D. Loss, "Spin relaxation and anticrossing in quantum dots: Rashba versus dresselhaus spin-orbit coupling," Phys. Rev. B **71**, 205324 (2005).

[7] A. Sammak, D. Sabbagh, N. W. Hendrickx, M. Lodari, B. Paquelet Wuetz, A. Tosato, L. Yeoh, M. Bollani, M. Virgilio, M. A. Schubert, P. Zaumseil, G. Capellini, M. Veldhorst, and G. Scappucci, "Shallow and undoped germanium quantum wells: A playground for spin and hybrid quantum technology," Advanced Functional Materials **29**, 1807613 (2019).

[8] N. W. Hendrickx, L. Massai, M. Mergenthaler, F. J. Schupp, S. Paredes, S. W. Bedell, G. Salis, and A. Fuhrer, "Sweet-spot operation of a germanium hole spin qubit with highly anisotropic noise sensitivity," Nature Materials (2024), 10.1038/s41563-024-01857-5.

[9] M. Brickson, N. T. Jacobson, A. J. Miller, L. N. Maurer, T.-M. Lu, D. R. Luhman, and A. D. Baczewski, "Using a high-fidelity numerical model to infer the shape of a few-hole ge quantum dot," (2024), `https://arxiv.org/abs/2408.14422` (accessed 2026-04-21).

[10] N. W. Hendrickx, D. P. Franke, A. Sammak, G. Scappucci, and M. Veldhorst, "Fast two-qubit logic with holes in germanium," Nature **577**, 487 (2020).

[11] C.-A. Wang, V. John, H. Tidjani, C. X. Yu, A. S. Ivlev, C. Déprez, F. van Riggelen-Doelman, B. D. Woods, N. W. Hendrickx, W. I. L. Lawrie, L. E. A. Stehouwer, S. D. Oosterhout, A. Sammak, M. Friesen, G. Scappucci, S. L. de Snoo, M. Rimbach-Russ, F. Borsoi, and M. Veldhorst, "Operating semiconductor quantum processors with hopping spins," Science **385**, 447 (2024).

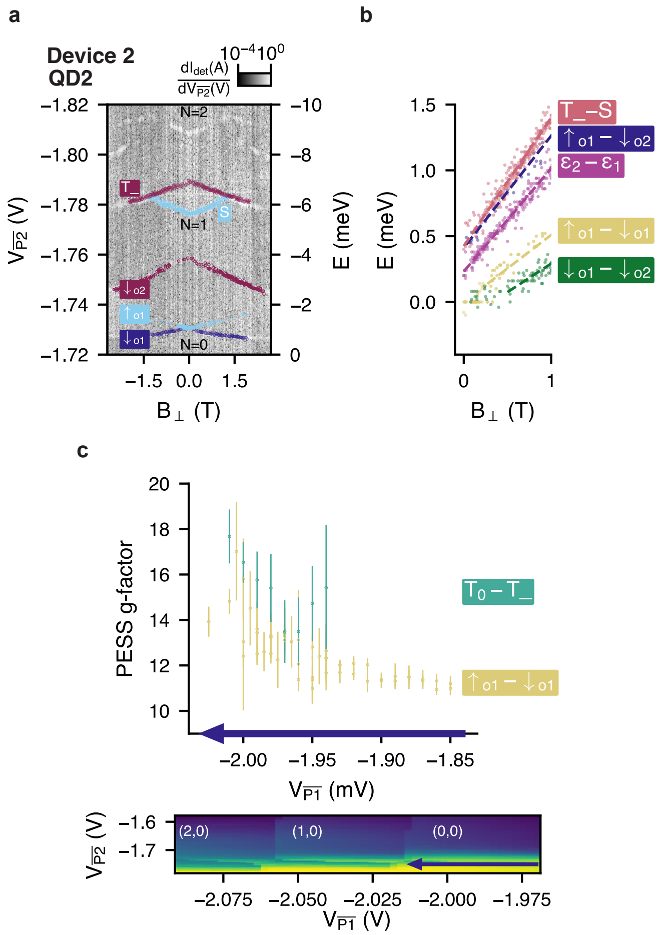

Figure S14. **Pulsed excited-state spectroscopy and g-factor extraction for QD2.** **(a)** PESS measurement of QD2. $\mathrm{d}I_{\mathrm{det}}/\mathrm{d}V_{\overline{\mathrm{P1}}}$ as a function of $V_{\overline{\mathrm{P2}}}$ and $B_{\perp}$ at fixed $\delta V_{\overline{\mathrm{P2}}}$. The hole number N is labeled, and regions sensitive to excited-state spectra are highlighted. Extracted energy levels are overlaid as colored lines. **(b)** Extracted spin-split energy pairs from PESS are analyzed to determine the CBAS g-factor for hole occupations $N = 1$ and $N = 2$, following the same procedure as in the CBAS method. Additionally, PESS g-factors are extracted from the spin-split excited-state transitions including orbital contribution ($\downarrow_{\mathrm{o1}}$–$\downarrow_{\mathrm{o2}}$, $\uparrow_{\mathrm{o1}}$–$\downarrow_{\mathrm{o1}}$, T_{-}–S). **(c)** PESS g-factor of QD2 as a function of the plunger gate voltage applied to the neighboring quantum dot. The lower plot shows the corresponding charge stability diagram, with arrows indicating the direction of plunger gate tuning. The charge configuration is labeled as (N, M).

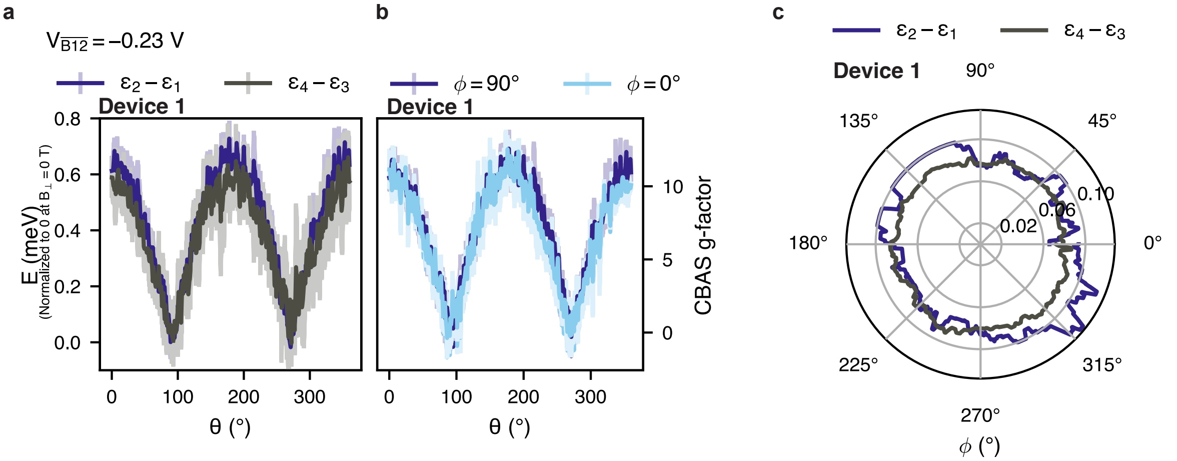

Figure S15. **Tilted magnetic field magnetospectroscopy and CBAS g-factor anisotropy.** Measurements are performed at $V_{\overline{\mathrm{B12}}} = -0.23\,\mathrm{V}$ and $|B| = 1\,\mathrm{T}$. The CBAS g-factor is extracted by subtracting spin-split energy levels and fitting the slope from the origin ($B = 0\,\mathrm{T}$, spin splitting $= 0$) for two spin pairs: $(\epsilon_2 - \epsilon_1)$ and $(\epsilon_4 - \epsilon_3)$. **(a)** CBAS g-factor anisotropy as a function of hole number at fixed azimuthal angle $\phi = 0°$. **(b)** CBAS g-factor anisotropy for two different values of ϕ for the $N = 1 \leftrightarrow 0$ transition. **(c)** In-plane CBAS g-factor for different hole numbers measured at polar angle $\theta = 90°$.

Panels a and b share the same y-axes for direct comparison.

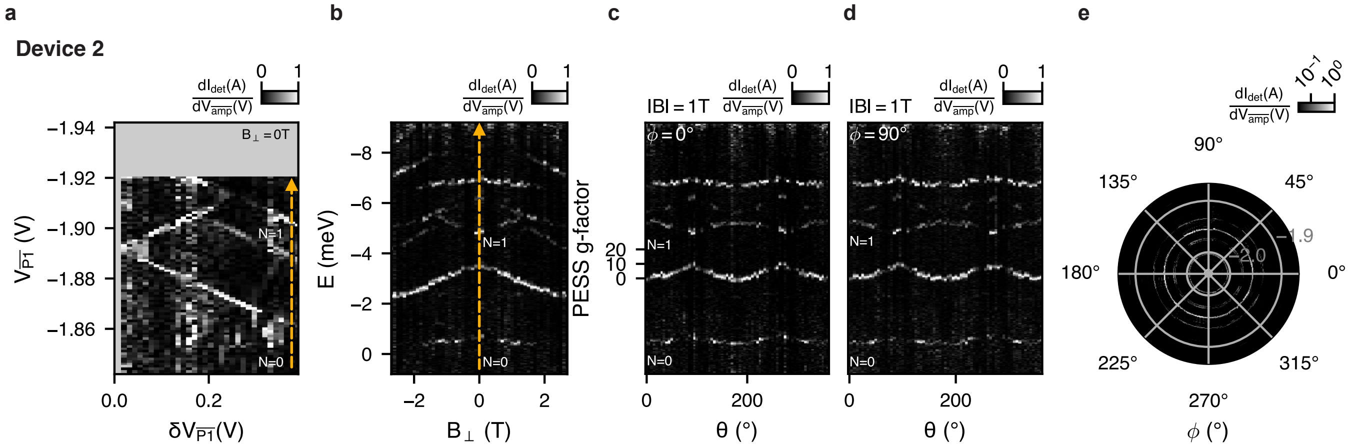

Figure S16. **PESS in dependence of tilted magnetic field.** **(a)** Derivative of the charge sensor current, $|dI_{\mathrm{SD}}/dV_{\overline{\mathrm{P1}}}|$, plotted as a function of pulse amplitude $\delta V_{\overline{\mathrm{P1}}}$ and dc gate voltage $V_{\overline{\mathrm{P1}}}$ at $B_\perp = 0$. **(b)** $|dI_{\mathrm{SD}}/dV_{\overline{\mathrm{P1}}}|$ as a function of $V_{\overline{\mathrm{P1}}}$ and $B_\perp$ at fixed $\delta V_{\overline{\mathrm{P1}}}$. Hole number N is indicated. **(c)** PESS g-factor anisotropy measured at $|B| = 1\,\mathrm{T}$ for azimuthal angle $\phi = 0°$. The g-factor is extracted from the slope of the spin-split energy versus magnetic field, extrapolated to the origin ($B = 0\,\mathrm{T}$, spin splitting $= 0$). The y-axis is consistent with panels **a** and **b**. **(d)** Same as panel **c**, but for $\phi = 90°$. **(e)** In-plane anisotropy of the PESS g-factor measured at polar angle $\theta = 0°$. Labels indicate the corresponding $V_{\overline{\mathrm{P1}}}$ values.

Panels (a–d) share the same y-axes for direct comparison.
